# Influence of sub-zero temperature on nucleation and growth of copper nanoparticles in electrochemical reactions

**DOI:** 10.1016/j.isci.2021.103289

**Published:** 2021-10-15

**Authors:** Qiubo Zhang, Jiawei Wan, Junyi Shangguan, Sophia Betzler, Haimei Zheng

**Affiliations:** 1 Materials Science Division, Lawrence Berkeley National Laboratory, Berkeley, CA 94720, USA; 2 Department of Materials Science and Engineering, University of California, Berkeley, CA 94720, USA

**Keywords:** Catalysis, Nanoparticles, Electrochemical materials science, Materials science, Materials characterization techniques

## Abstract

Cu metal nanostructures have attracted wide interest of study as catalysts for CO_2_ reduction reaction and other applications. Controlling the structure and morphology of Cu nanostructures during synthesis is crucial for achieving desired properties. Here, we studied temperature effects on electrochemical deposition of Cu nanoparticles. We found the size, nucleation density, and crystallinity of Cu nanoparticles are strongly influenced by low temperature processing. The electrodeposition at low temperature (−20°C) results in clusters of assembled small Cu nanoparticles, which is distinctly different from the large individual highly crystalline Cu nanoparticles obtained from the room temperature process. The differences in Cu nanoparticle morphology and crystallinity are attributed to the variations in reduction reaction rate and surface diffusion. The limitation of the reaction rate promotes multiple nuclei, and low surface diffusion induces poor crystallinity. This study deepens our understanding of low-temperature effects on electrochemical processes assisting the design of diverse hierarchical catalytic materials.

## Highlights

• Achieve two kinds of hierarchical copper nanoparticle/nanowire nanostructures
• Low-temperature changes the growth mode from diffusion-limited to reaction-limited
• At low-temperature, low surface diffusion induces poor crystallinity of copper

## Graphical abstract

**Figure:**
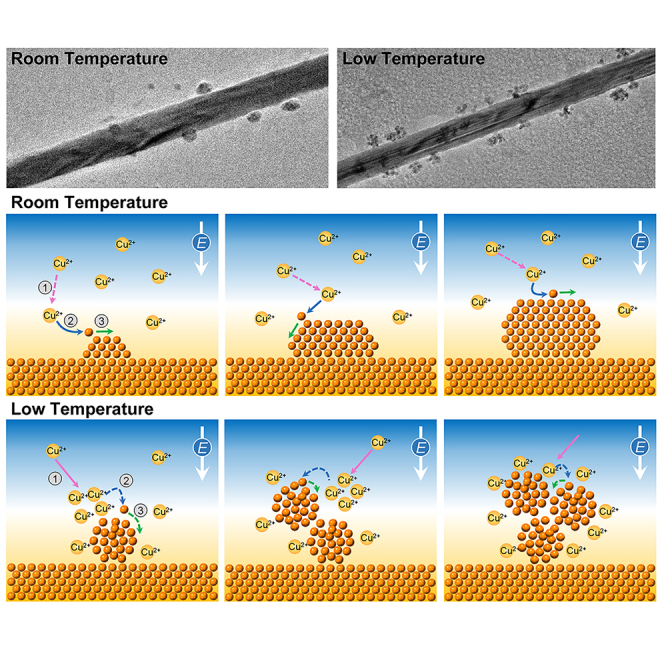

## Introduction

Copper-based materials have emerged as exciting catalysts for electrochemical carbon dioxide reduction reactions (CO_2_RR), which converts CO_2_ into renewable fuels and feedstock (Birdja et al., 2019; Choi et al., 2020; Kim and Palmore, 2020; Nitopi et al., 2019; Ross et al., 2019). A variety of multi-carbon products can be achieved during CO_2_RR, such as ethylene, ethanol and propanol (Gattrell et al., 2006). The efficiency and selectivity of these catalysts are dependent on the structure and morphology of the Cu catalysts, e.g., the nanoparticle sizes (Li et al., 2016; Stephens et al., 2012), configurations (Grosse et al., 2018), crystallinity (Li et al., 2018) and oxidation states (De Luna et al., 2018). Therefore, understanding and controlling of Cu nanomaterials formation during synthesis is essential in order to optimize their catalytic performance.

Compared to many other methods, electrodeposition avoids the high temperature synthetic procedures, thus it has been considered as an ideal way for the preparation of hierarchically structured catalysts (Che et al., 2018; Lu and Zhao, 2015). In electrodeposition, there are many factors that may affect the nucleation and growth of nanocrystals, and result in diverse structure and morphology of the final products (Day et al., 2007). For example, the current density can affect the nucleation density, size and shape of the deposited nanocrystals (Pei et al., 2017), whereas the pH value affects the structure and properties of the metal deposits (Kumar et al., 2015). Additives in the solution can inhibit the formation of lithium dendrites during battery operation (Lee et al., 2020). Although there have been reports on temperature impacts on electrodeposition (Qiao et al., 2013; Yan et al., 2019), it is not well understood how low temperature affects the nucleation and growth of Cu nanoparticles. This may arise from two main reasons. First, it is difficult for general electrolytes to maintain a liquid state and good electrical conductivity at low temperatures. Second, it is challenging to characterize the products because they may change after being removed from the low temperature environment.

Here, we synthesize hierarchical Cu nanostructures with Cu nanoparticles attached to the Cu nanowires at both sub-zero temperature (−20°C) and room temperature (20°C). The Cu nanostructures are characterized using transmission electron microscopy (TEM) with different techniques, including liquid cell electron microscopy, cryo-EM, etc. We found that sub-zero temperature impacts the crystallinity and morphology of the Cu nanoparticles. The different growth modes of Cu nanoparticles at the low temperature, such as nucleation and assembly of multiple small Cu nanoparticles to form Cu nanoclusters, are attributed to the reduced reaction rate. The low surface diffusion rate of adsorbed atoms at the low temperature is considered as the main factor responsible for the poor crystallinity.

## Results and discussion

### Experimental setup

**Figure 1:**
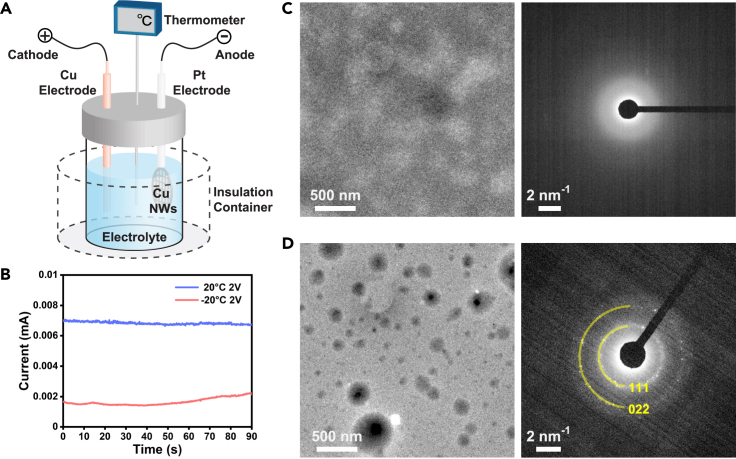
Experimental setup and characterization of electrolyte at room temperature (20°C) and sub-zero temperature (−20°C) (A) A schematic diagram of experimental setup. (B) Current vs. time plots of the electrodeposition processes at different temperatures. (C) TEM and electron diffraction images of the electrolyte in a carbon film liquid cell at 20°C. (D) TEM and electron diffraction images of the electrolyte in a carbon film liquid cell at −20°C. Solid precipitations are found in the electrolyte.

We conducted the experiments with a well-designed device to realize electrochemical and sub-zero temperature control. The schematic illustration of the experimental setup is shown in Figure 1A. First, Cu nanowires were prepared in advance and loaded onto a carbon film-supported TEM copper grid (see STAR Methods). Then, the copper grid was connected to the platinum anode, and the cathode was made with copper. The two electrodes were connected to an electrochemical workstation and immersed into a 5 mL commercial electrolyte of 1.0 M LiPF_6_ in ethylene carbonate/diethyl carbonate (EC/DEC = 50/50 (v/v)). We choose EC/DEC mixed solution as the electrolyte because of its lower freezing point compared to aqueous solution (Ding et al., 2001), which helps to maintain a liquid state and good electrical conductivity even at sub-zero temperature. After that, we sealed the electrolyte in a ceramic container surrounded by an insulating device made of polystyrene to control the temperature. Liquid nitrogen was used to cool down the insulation device to a desired sub-zero temperature, at which can be maintained for a few minutes because of the low thermal conductivity of polystyrene (0.033 W/(m·K)) (Canetta et al., 2014).

In the experiments, we use constant voltage operations at 2 V for 90 s for experiments at room temperature (20°C) and sub-zero temperature (−20°C). The temperature was measured in real time with an electric thermal detector. As shown in Figure 1B, the current is stable at 0.007 mA at 20°C and 0.002 mA at −20°C. Current density is lower at the low temperature because ion migration slows down in the electrolyte.

To further find out the differences in electrolyte structure at different temperatures, we used liquid-phase TEM combined with cryo-EM techniques to image electrolytes at 20°C and −20°C. TEM images and selected area electron diffraction (SAED) patterns of the electrolyte at 20°C and −20°C are shown in Figures 1C and 1D. We loaded a droplet of electrolyte (1.0 M LiPF_6_ in EC/DEC = 50/50 (v/v)) on one carbon film and it was sandwiched by another carbon film to form a thin liquid cell. At room temperature, no diffraction spot is found in the diffraction pattern which suggests the electrolyte presents a homogeneous liquid structure. To achieve the low temperature for liquid cell TEM imaging, we used the cryo-EM sample stage that can be cooled down and stabilized at −20°C. The TEM image shows phase separation of the electrolyte at −20°C, which consists of both liquid and solid phases. The corresponding diffraction spots match the {111} and {022} crystal facets of solid EC. At low temperatures, solid EC precipitates out of the liquid phase electrolyte and the electric conductivity of the electrolyte decreases.

### Structural characterization of the as-prepared Cu nanowires

**Figure 2:**
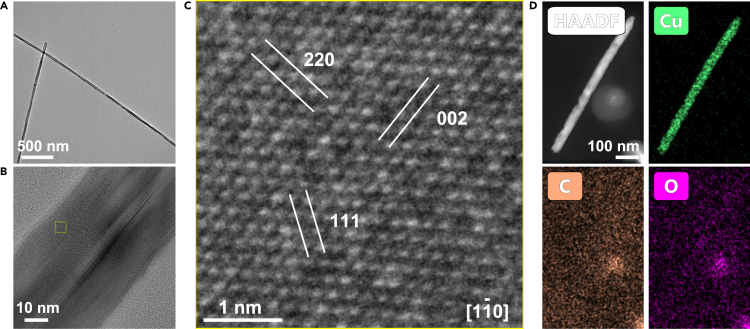
Structural characterization of the as-prepared Cu nanowires (A) Low magnification TEM image of the Cu nanowires. (B) HRTEM image of one Cu nanowire. (C) The zoom-in image of the yellow box area in (B) showing the crystal orientation of the nanowire. The zone axis is $\left[1\bar{1}0\right]$. (D) STEM-EDX elemental mapping of Cu nanowire: HAADF image, Cu map, C map and O map. See also Figures S1 and S2.

We characterized the as-prepared Cu nanowires to facilitate the comparison of samples before the electrodeposition (Figure 2). The synthesized Cu nanowires typically display a fivefold twin with the <110 > axial direction and {100} side facets (Choi et al., 2020; Jin et al., 2011). A low magnification TEM image demonstrates its one-dimensional wire structure with an average diameter of about 30 nm (Figures 2A and S1). The length of the Cu nanowires ranges from hundreds of nanometers to several micrometers. Figure 2B is the high-resolution TEM (HRTEM) image of the Cu nanowires. The atomic resolution image (Figure 2C) is enlarged from the inset box in Figure 2B. It shows the lattice spacing of Cu {111}, {002} and {200}, which is consistent with the <110> axial-growth direction of the Cu nanowires. In addition, the FFT pattern of the yellow square area shows Cu single crystalline structure (Figure S2). High-angle annular dark-field (HAADF) STEM image and energy dispersive X-ray (EDX) mapping (Figure 2D) also confirms the successful synthesis of Cu nanowires. We focus on chemical mapping of the distribution of copper (Cu), carbon (C), and oxygen (O), which shows nanowires of pure copper without noticeable oxidation.

### Structural diversity of Cu nanoparticles affected by temperature

**Figure 3:**
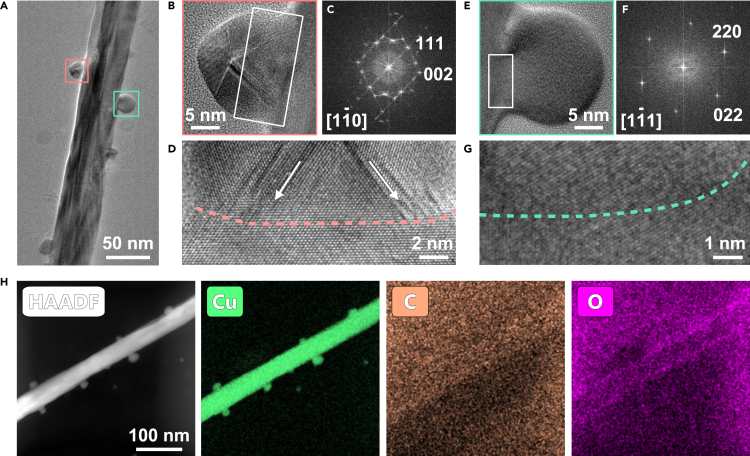
Structure characterization of the hierarchical Cu nanoparticle/nanowire nanostructure formed at room temperature (A) Low magnification TEM image of the hierarchical Cu nanoparticle/nanowire nanostructure. (B) The zoom-in image of the pink box in (A) shows the nanoparticle with a five-fold twin structure. (C) The corresponding FFT pattern of (B). (D) The zoom-in image of the white box area in (B) showing the interface between nanoparticle and nanowire. The pink dotted line marks out the interface. (E) The zoom-in image of the green box in (A) shows the nanoparticle with a single crystal structure. (F) The corresponding FFT pattern of (E). (G) The zoom-in image of the white box area in (E) showing the interface between single crystal nanoparticle and nanowire. The green dotted line marks out the interface. (H) STEM-EDX elemental mapping of hierarchical Cu nanoparticle/nanowire nanostructure: HAADF image, Cu map, C map and O map. See also Figures S3, S4, S6, and S7.

After electrodeposition, we obtained two kinds of hierarchical structures depending on the temperature and named them Cu hierarchical nanoparticle/nanowire (NP/NW) structure and nanocluster/nanowire (NC/NW) structure. Electrodeposition at room temperature (20°C) produces individual nanoparticles attached to the nanowire, forming NP/NW hierarchical structure, as shown in Figure 3A. Although the sizes of these nanoparticles are not uniform spanning from 10 nm to 22 nm, the average diameters are about 16 nm (Figure S3). The HRTEM images show that these nanoparticles have good crystallinity. According to their crystal structures, they can be divided into five-fold twin crystals and single crystals (Figures 3B and 3E). The HRTEM image and the corresponding FFT pattern of the five-fold twin show that the twin boundaries belong to (111) crystal facets (Figure 3C). An enlarged image of the interface between nanoparticles and nanowires (Figure 3D) shows that there is no obvious dislocation or lattice distortion at the interface. Strain located at the twin boundary of the five-fold twin can be found, which might be because of the slight changes in lattice orientation.

The single-crystal particles are almost spherical. The corresponding FFT pattern shows that there is only one set of diffraction spots, and the crystal structures of nanoparticle and nanowire substrate perfectly match (Figure 3F). The enlarged image (Figure 3G) of the interface further confirms that the nanoparticles and nanowires are integrated, with no detectable differences in the lattice structures. The EDX spectrum verified that the particles only contain copper elements and the appearance of the C peak is caused by the carbon film support (Figure S4). Figure 3H shows the distribution of the elements. Copper is evenly distributed in the material.

**Figure 4:**
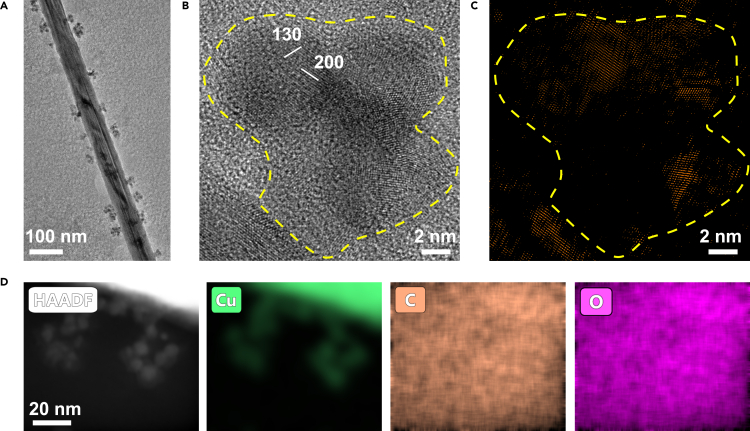
Structure characterization of the hierarchical Cu nanocluster/nanowire nanostructure formed at −20°C (A) Low magnification TEM image of the hierarchical Cu nanocluster/nanowire nanostructure. (B) HRTEM image of several gathered Cu nanoclusters shows the crystallinity of the clusters is not perfect. (C) The inverse fast Fourier transform (IFFT) of the HRTEM image highlights the crystal part. The yellow dotted line marks the edge of the cluster. (D) STEM-EDX elemental mapping of the hierarchical Cu nanocluster-nanowire nanostructure: HAADF image, Cu map, C map, and O map. See also Figures S5–S7.

In contrast, at −20°C, the morphology of Cu deposits are completely different and Cu clusters are found. Because copper is an active metal under electron beam irradiation (Lin et al., 2019), to avoid potential alteration by electron beam irradiation during imaging, cryogenic TEM imaging was adopted. As shown in Figure 4A, we find that the deposited Cu tends to form nanoclusters rather than individual nanoparticles. These nanoclusters are the assembly of smaller nanoparticles and they connect with the nanowire forming hierarchical Cu NC/NW nanostructure. Compared with nanoparticles formed at room temperature, the nanoclusters are smaller in diameter (about 9 nm), and the nucleation density of nanoclusters is greater with the assembled smaller Cu nanoparticles. HRTEM image and the corresponding FFT pattern reveal the poor crystallinity of the nanoclusters (Figures 4B and S5). Within a cluster, both crystalline regions and disordered regions are found. The crystalline part of the cluster is highlighted by performing the inverse Fourier transform of the diffraction spots in the FFT. Subsequently, the image showing the crystalline regions was dyed with orange color. As shown in Figure 4C, the aggregation of nanoparticles within a nanocluster has no preferred orientation. The FFT pattern of Cu nanoclusters obtained from Figure 4B also shows randomly oriented polycrystalline Cu clusters (Figure S5). We can also find that the neck between the nanoclusters and the nanowires is narrower. Combining with the observed disordered structure, it implies that the interfacial bonding is weak. The EDX mapping in Figure 4D shows that only Cu exists in the sample. In summary, compared with the NP/NW structure formed at room temperature, the Cu NC/NW nanostructure formed at low temperature shows smaller sizes, the clusters of assembled small Cu nanoparticles, reduced crystallinity and weakened connection to the Cu nanowire.

### Electrochemical mechanism of nanoparticle growth at various temperatures

Regarding the size of Cu nanoparticles, previous studies have shown that both temperature and current density impact the size of the deposited particles in electrochemical processes by changing the overpotential (Ely and García, 2013; Pei et al., 2017). Classical nucleation equations can be used to understand the dependence of Cu particle sizes on the overpotential. The critical radius (*r*_*crit*_) of nucleation can be described as it follows (Ely and García, 2013; Pei et al., 2017):

(Equation 1) $$r_{crit}=2ϒV_{m}/F\left|\text{η}\right|$$

Where F is Faraday's constant, ϒ is the surface energy of the Cu-electrolyte interface, $V_{m}$ is the molar volume of Cu, and η is the electrochemical overpotential. This equation is also applicable to express the critical nuclei size in heterogeneous nucleation (Ely and García, 2013; Sano et al., 2014). Obviously, the nuclei size is inversely proportional to the electrochemical overpotential. It has been demonstrated in previous studies that the nucleation overpotential increases at lower temperatures (Yan et al., 2019), resulting in smaller metal nuclei. Furthermore, as predicted by the Butler−Volmer electrode kinetics relationship between the current density and electrode potential, the current density decreases as the nucleation overpotential decreases (Ely and García, 2013). Thus, under low current density conditions at the low temperature, the nuclei sizes are expected to be larger. However, in our experiment, the nucleation size is smaller corresponding to a low current density at −20°C. (Figures S6 and S7). Therefore, we conclude that the effect of temperature on the nucleation overpotential is larger than the influence of the current density.

To understand the differences in particle aggregation, interfacial bonding and crystallinity, we analyzed the entire process. The metal electrodeposition process usually includes three steps (Paunovic and Schlesinger, 2006; Saidin et al., 2020). For the first step, previous studies have proved that low temperature can reduce the ion diffusion coefficient (Roy et al., 2017). Similarly, nucleation overpotential increases with the decreasing temperature (Yan et al., 2019), the electroreduction reaction at the solid-liquid interface is more difficult to take place. Regard to the third step, according to the surface diffusion theory, the diffusion coefficient D is given by (Antczak and Ehrlich, 2007):

(Equation 2) D=(ve−EdiffKBT)a2/z

Where *v* is attempt frequency, T is the absolute temperature, $K_{B}\;$ is Boltzmann constant, *E*_*diff*_ is the potential energy barrier for diffusion, and a is the distance per jump. z = 2 for one dimensional diffusion, z = 4 for two dimensional diffusion, and z = 6 for three dimensional diffusion.

**Figure 5:**
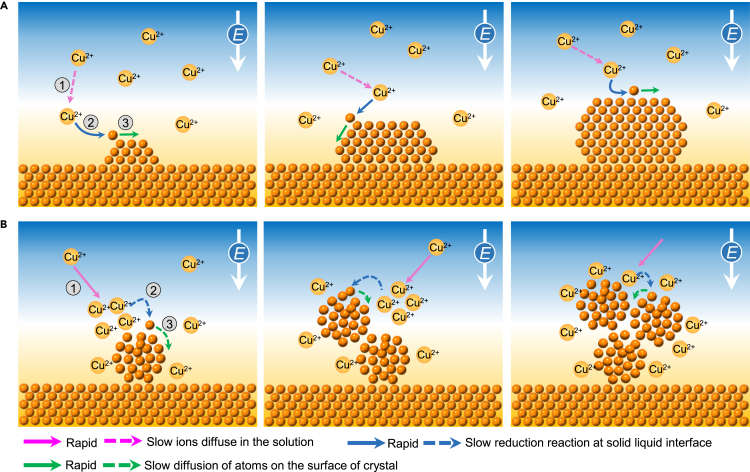
Schematic illustrations of two kinds of electrochemical growth mechanisms of copper metal (A) The growth of individual large crystalline Cu nanocrystals is limited by the diffusion of ions in the solution at room temperature. (B) The growth of Cu nanoclusters with smaller and weaker interfaces is limited by the reduction reaction at the electrolyte/metal interface at −20°C.

At room temperature, Cu electrodeposition is limited by the diffusion of ions in the solution. As shown in Figure 5A, the Cu ions in the solution diffuse to the electrolyte/electrode interface under the electric bias, then reduced to adsorbed Cu atoms. The newly-born adsorbed metal atoms diffuse along the nanowire surface and aggregate with other newly-born atoms to form nanocrystals. Because the surface diffusion of Cu atoms is relatively fast at room temperature, they can find the lowest surface energy sites, resulting in the epitaxial formation of independent single crystal nanoparticles on the nanowires.

At low temperatures (Figure 5B), although the rate of all three steps will decrease, the decrease of the reaction rate is more serious than the diffusion rate. Therefore, the whole process becomes limited by the reaction rate. The Cu ions diffuse to the liquid/solid interface and are reduced to adsorbed atoms. The first formed adsorbed atoms cannot diffuse rapidly resulting in a small contact area between nanoclusters and nanowires. The following ions cannot be reduced in time because of the restriction of the reaction, so they will gather near the surface of the nanoclusters and nucleate again to form a new cluster after the concentration reaches a critical value. Owing to the surface diffusion limitation of the adsorbed atoms, the newborn surface atoms can't reach the lowest energy positions and are randomly fixed at the reaction sites, resulting in poor crystallinity. This qualitatively explains the observed Cu nanocluster formation at the sub-zero temperature.

### Conclusion

In conclusion, we have demonstrated the temperature-dependent structure diversity of copper nanoparticles from electrodeposition. The electrodeposition at sub-zero temperature leads to the growth of Cu clusters with smaller Cu nanoparticles. The particle size, morphology, crystallinity and connection to the substrate (Cu nanowire) are distinctly different from those obtained at room temperature. The reduced reaction rate results in multi-nuclei and weak bonding at the interfaces. The decreased surface diffusion rate at the low temperatures may also induce poor crystallinity of Cu clusters. This study deepens our understanding of nucleation in nanostructures and sheds light on the governing factors in the electrodeposition of metal nanoparticles and nanoscale dynamic processes.

### Limitations of the study

In this study, the sub-zero temperature results in a diversity of the electrodeposited copper structures. These three steps: the diffusion of copper ions in the solution, the oxidation-reduction reaction at the solid-liquid interface, and the diffusion of copper ions on the surface of the particles, determine the final structure of the particles. Here, we provide a method to optimize the structure of electrodeposited metals by sub-zero temperature and discover the underlying mechanism. However, the final structure of different metals should be diverse that formed at different temperatures, and the specific differences are determined by the three sub-processes and the nature of the metal. Therefore, different metals have different degrees of temperature dependence, and the temperature-dependent structural differences may not be the same for other metals.

## STAR★Methods

### Key resources table

**Table:** 

REAGENT or RESOURCE	SOURCE	IDENTIFIER
**Chemicals, Peptides, and Recombinant Proteins**		

Copper (II) chloride (CuCl_2_, 99.999%)	Sigma-Aldrich	CAS#7447-39-4
D-(+)-glucose (≥99.5%)	Sigma-Aldrich	CAS#50-99-7
Ethanol (≥99.0%)	Sigma-Aldrich	CAS#64-17-5
Hexane (95%)	Sigma-Aldrich	CAS#110-54-3
Lithium hexafluorophosphate solution (1 M lithium hexafluorophosphate (LiPF_6_) in ethylene carbonate (EC) and dimethyl carbonate (DEC) solution (volume ratio 1:1))	Sigma-Aldrich	MDL#MFCD00011096

**Softwareand Algorithms**		

EC-lab V11.27	BioLogic Science Instruments	https://www.biologic.net/
ImageJ	ver 2.1.0/1.53c	https://imagej.net/

**Other**		

SP-200 potentiostat	BioLogic Science Instruments	https://www.biologic.net/
Themis TEM	Thermo Fisher Scientific	https://www.fei.com/

### Resource availability

#### Lead contact

Further information and requests for resources should be directed to and will be fulfilled by the lead contact: Haimei Zheng (hmzheng@lbl.gov).

#### Materials availability

All materials generated in this study are available from the lead contact without restriction.

### Method details

#### Preparation of Cu nanowires

In a standard synthesis of the Cu nanowires, 17 mg of CuCl_2_, 50 mg of D-(+)-glucose and 180 mg of hexadecylamine were mixed with 10 mL of deionized water in a glass vial. The final solution was sonicated for 30 min at room temperature. The vial was then transferred into an oil bath and heated at 100°C for 6 h under magnetic stirring. The synthesized Cu nanowires were washed five times with hexane/ethanol (1:1 volume) and collected by centrifuge at 9500 rpm for 5 min.

#### TEM imaging

The TEM experiments were performed on a FEI ThemIS aberration-corrected TEM at the Molecular Foundry (MF), Lawrence Berkeley National Laboratory (LBNL). The microscope was operated at 300 keV with a Super-X energy dispersive X-ray spectroscopy (EDS) detector, allowing for rapid chemical identification. The cryo-EM imaging was performed by using a Gatan 915 cryo-transfer holder.

### Quantification and statistical analysis

We counted 20 particles generated at room temperature and −20°C and measured the particle sizes with ImageJ software. The size distribution is shown in Figure S7. We further calculated the mean value and standard deviation, as shown in Figure S6.

## Data Availability

• All data reported in this paper will be shared by the lead contact upon request. • This paper doesn't report original code. • Any additional information required to reanalyze the data reported in this paper is available from the lead contact upon request.
