# Supplementary material for: Influence of sub-zero temperature on nucleation and growth of copper nanoparticles in electrochemical reactions
Source: iScience. 2021 Oct 15;24(11):103289. doi: 10.1016/j.isci.2021.103289 (PMC8577071; doi:10.1016/j.isci.2021.103289)
Supplement: Document S1. Figure S1–S7 [file mmc1.pdf]

iScience, Volume *24*

Supplemental information

Influence of sub-zero temperature on nucleation and growth of copper nanoparticles in electrochemical reactions

Qiubo Zhang, Jiawei Wan, Junyi Shangguan, Sophia Betzler, and Haimei Zheng

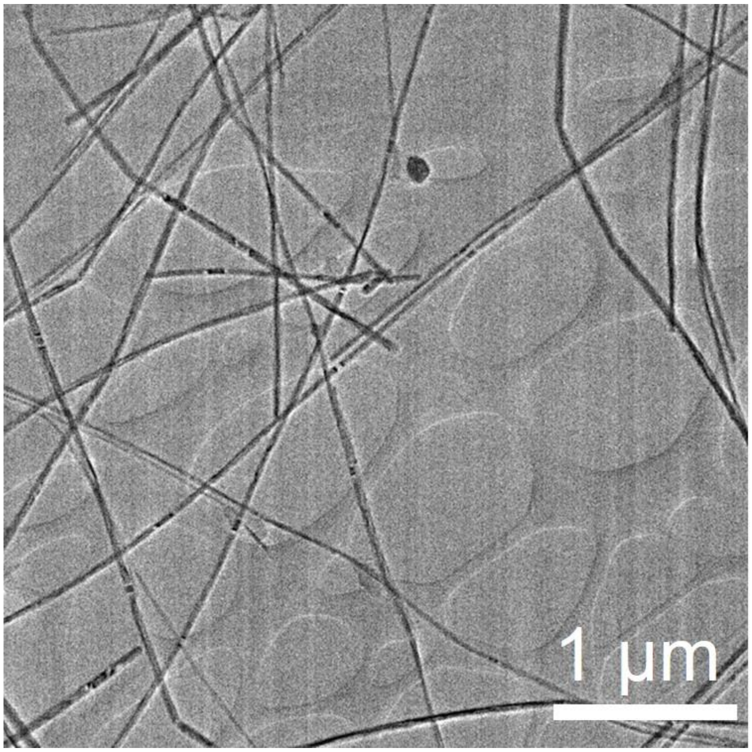

Figure S1. Low resolution TEM image of the Cu nanowires. Related to Figure 2.

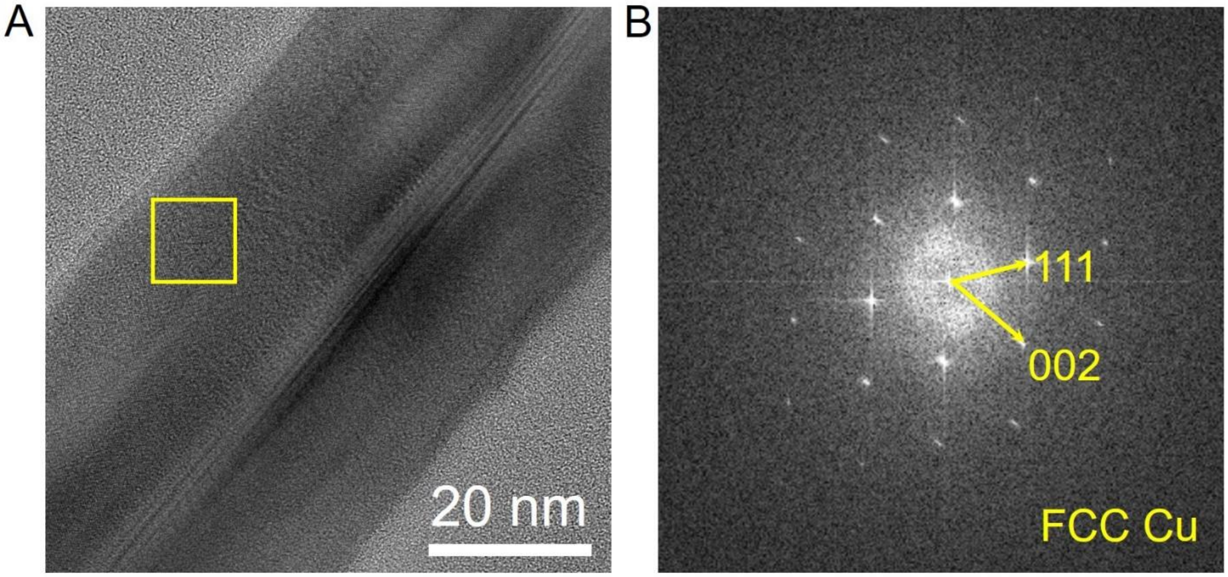

Figure S2. (A) HRTEM image of one nanowire. (B) FFT pattern of as prepared Cu nanowire from the yellow square area showing single crystalline structure. Related to Figure 2.

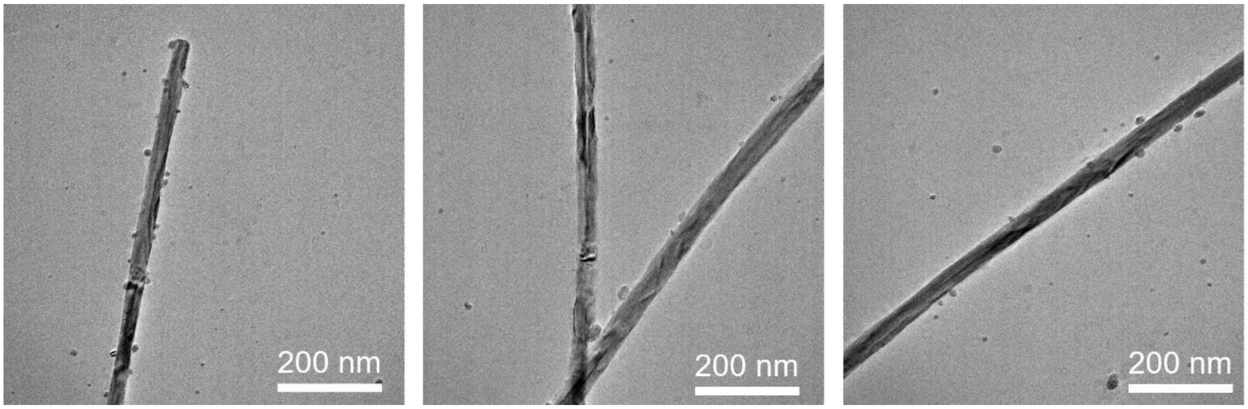

Figure S3. Low resolution TEM image of the hierarchical Cu nanoparticle-nanowire nanostructure. Related to Figure 3.

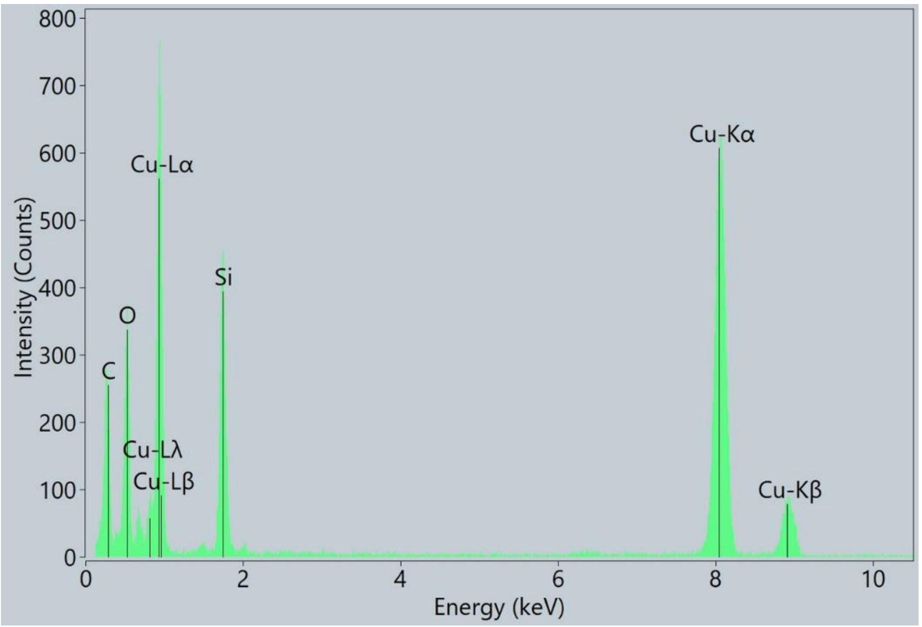

Figure S4. EDX spectra of Cu nanostructures formed at room temperature with large crystalline Cu nanoparticles attached to the Cu nanowires. Related to Figure 3.

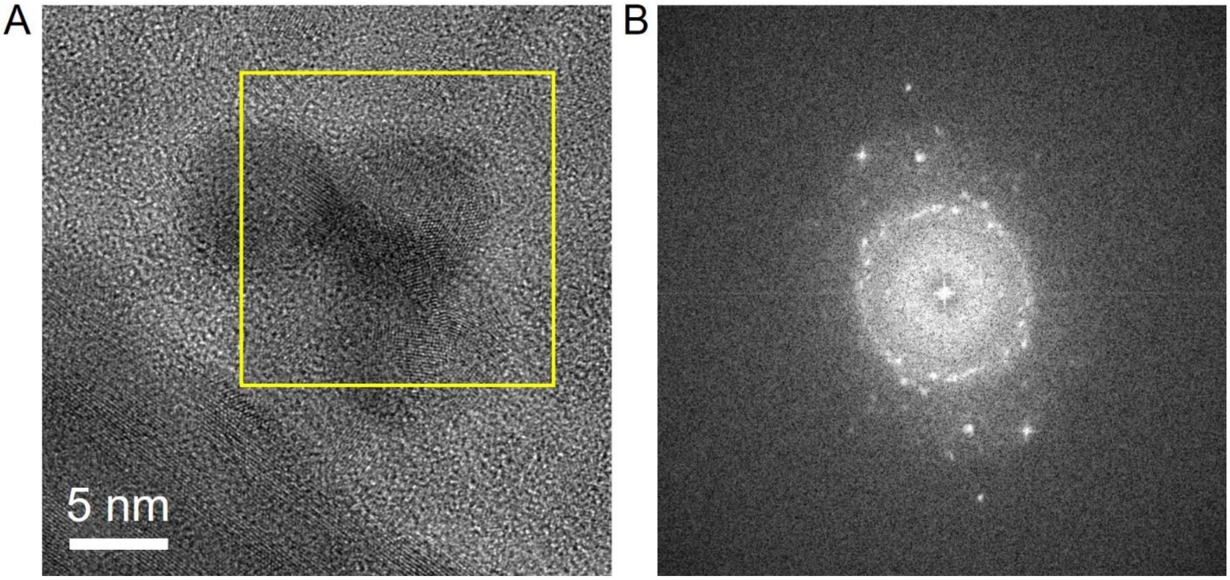

Figure S5. (A) TEM image of the hierarchical Cu nanocluster-nanowire nanostructure. (B) FFT pattern of Cu nanoclusters obtained from the yellow square area at low temperature (-20 ℃). It shows random oriented polycrystalline Cu clusters. Related to Figure 4.

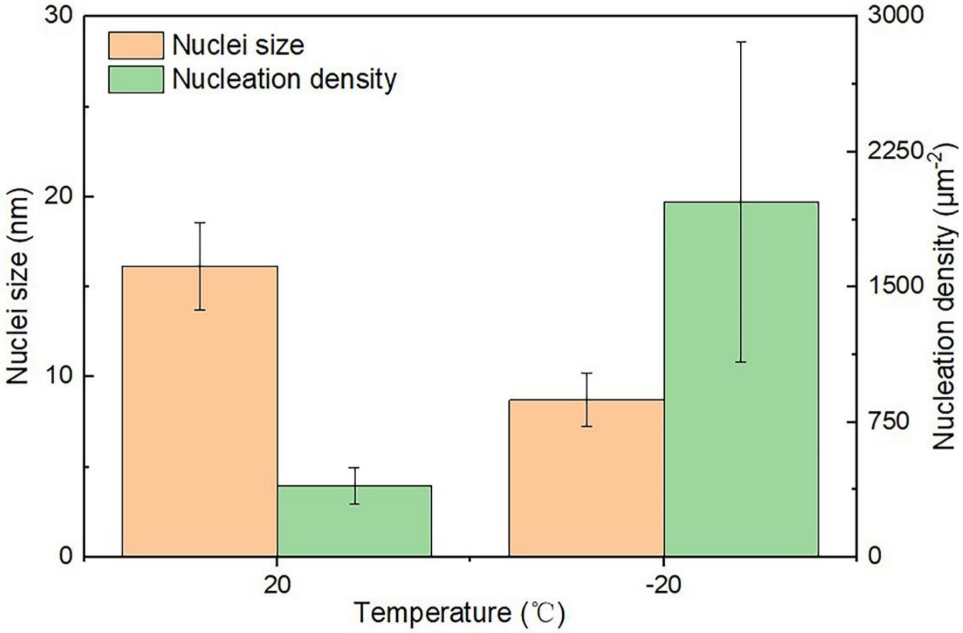

Figure S6. Nuclei size and nucleation density of Cu nanoparticles at 20 ℃ and -20 ℃. Related to Figure 3 and Figure 4.

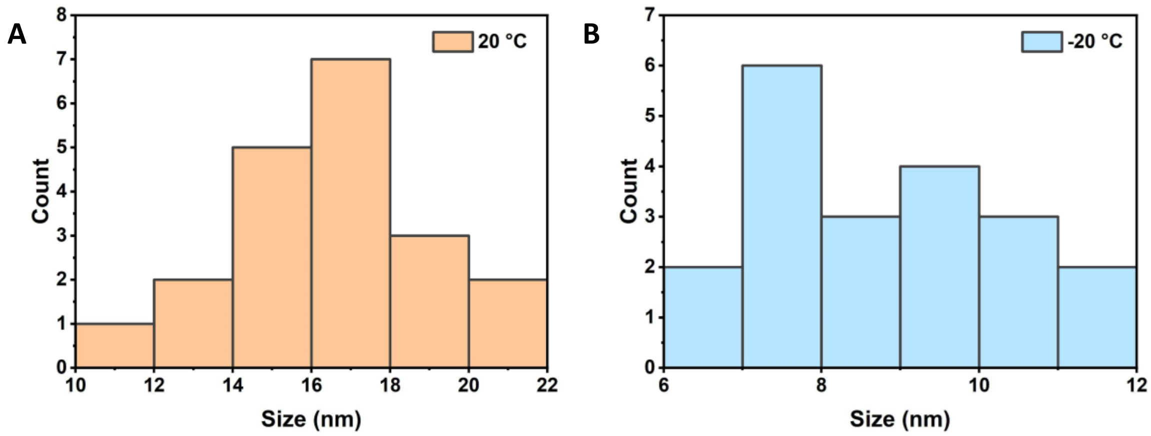

Figure S7. Histograms of Cu nanoparticles sizes at (A) 20 ^{o}C and (B) -20 ^{o}C. Related to Figure 3 and Figure 4.
